# Damping Characteristics of Ti_50_Ni_50−*x*_Cu*_x_* (*x* = 0~30 at.%) Shape Memory Alloys at a Low Frequency

**DOI:** 10.3390/ma7064574

**Published:** 2014-06-16

**Authors:** Chen Chien, Shyi-Kaan Wu, Shih-Hang Chang

**Affiliations:** 1 Department of Materials Science and Engineering, National Taiwan University, No. 1, Sec. 4, Roosevelt Road, Taipei 106, Taiwan; E-Mail: d01527004@ntu.edu.tw; 2 Department of Mechanical Engineering, National Taiwan University, No. 1, Sec. 4, Roosevelt Road, Taipei 106, Taiwan; 3 Department of Chemical and Materials Engineering, National I-Lan University, No. 1, Sec. 1, Shen-Lung Road, I-Lan 260, Taiwan; E-Mail: shchang@niu.edu.tw

**Keywords:** shape memory alloys, damping properties, martensitic transformation, twins, relaxation peak

## Abstract

The damping characteristics of Ti_50_Ni_50−*x*_Cu*_x_* (*x* = 0~30 at.%) shape memory alloys (SMAs) at a low frequency have been studied using a dynamic mechanical analyzer. The magnitude of the tan δ value and the values of the storage modulus (*E*_0_) softening/hardening and the strain variation exhibited in B2↔B19 transformation are all higher than those in B2↔B19’ transformation. The larger *E*_0_ softening/hardening in B2↔B19 can induce higher strain variation in this transformation. It is suggested that the greater mobility of the twin boundaries and the larger magnitude of the strain variation both cause the higher tan δ value exhibited in B2↔B19 transformation, as compared with B2↔B19’ transformation. In comparison with that in B19’ martensite, the *E*_0_ value in B19 martensite is low and not affected so greatly by changes in temperature. Relaxation peaks are observed in B19’ martensite, but not in B19 martensite, because the latter has rare twinned variants. The activation energy of the relaxation peak is calculated and found to increase as the Cu-content increases in these SMAs.

## 1. Introduction

TiNi shape memory alloys (SMAs), which undergo thermoelastic martensitic transformation can exhibit good shape memory effect (SME), pseudoelasticity (PE), and high damping capacity [1,2,3,4,5,6,7,8]. The addition of Cu into TiNi SMAs improves the SME and PE properties and reduces the temperature hysteresis of the SMAs [9,10]. It has been reported that the transformation sequences of Ti_50_Ni_50−*x*_Cu*_x_* (*x* = 0~30 at.%) SMAs are B2↔B19’, B2↔B19↔B19’ and B2↔B19, when the Cu-contents are *x* ≤ 7.5, 10 ≤ *x* ≤ 15 and 20 ≤ *x* ≤ 30, respectively [11,12,13]. The damping properties exhibited in TiNiCu SMAs can be examined with a dynamic mechanical analyzer (DMA). However, the phenomena of the storage modulus (*E*_0_) softening/hardening and the magnitude of the tan δ value exhibited in TiNiCu SMAs during B2↔B19’ and B2↔B19 martensitic transformations have not been clarified. In addition, a broad peak appears at around −70 °C in the DMA curve of TiNi/TiNiCu SMAs [14]. This broad peak, which does not correspond to *E*_0_ softening/hardening, is called the relaxation peak. However, the effect of the Cu-content of Ti_50_Ni_50−*x*_Cu*_x_* SMAs on the occurrence of the relaxation peak is not fully understood In this study, we examine the tan δ value and the *E*_0_ softening/hardening associated with B2↔B19’ and B2↔B19 transformations in Ti_50_Ni_50−*x*_Cu*_x_* (*x* = 0~30 at.%) SMAs. The damping properties exhibited in B19’ martensite are compared with those in B19 martensite. The effect of the Cu-content in Ti_50_Ni_50−*x*_Cu*_x_* SMAs on occurrence of the relaxation peak and its activation energy (*E*_a_) are also discussed.

## 2. Results and Discussion

### 2.1. Tan δ Value versus Temperature (T)

**Figure 1:**
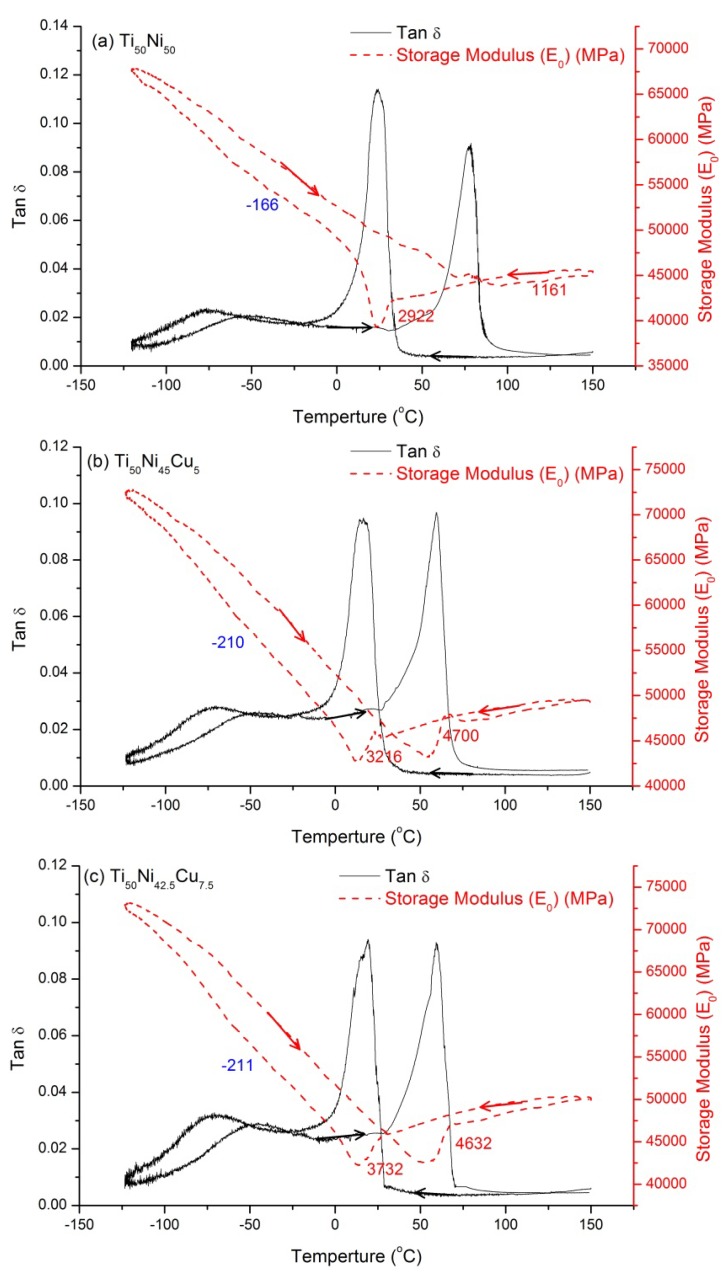
The tan δ value and storage modulus (*E*_0_) curves for Ti_50_Ni_50−*x*_Cu*_x_* SMAs. (**a**) *x* = 0; (**b**) *x* = 5; (**c**) *x* = 7.5 at.%. The blue number is the *E*_0_ slope in B19’ martensite, and the red numbers are the magnitude of *E*_0_ softening and hardening.

As mentioned in Section 2, DMA tests were conducted at a low frequency of 1 Hz and a cooling rate of 3 °C/min (instead at the isothermal condition, *i.e*., at 0 °C/min), thus, the obtained tan δ values are mostly contributed by the transitory term (IF_Tr_) [12,15,16]. Figure 1a–c show the tan δ value *versus* *T* curves for Ti_50_Ni_50−*x*_Cu*_x_* (*x* = 0, 5, 7.5 at.%) SMAs, respectively. According to the reported transformation sequence [11,12,13], Figure 1a indicates a B2→B19’ transformation peak appearing at 24 °C with a tan δ value of 0.11 in cooling, and a B19’→B2 transformation peak occurring at 78 °C with a tan δ value of 0.09 in heating. Figure 1b,c show the same transformation peaks as Figure 1a. The peak temperature and its tan δ value are found to decrease as the Cu-content increases. Figure 1 reveals that there is a transformation peak appearing in the cooling/heating curve which is regarded as a B2↔B19’ transformation exhibited in SMAs when the Cu-content is below 7.5 at.%. In addition, there are relaxation peaks at about −70 °C and −50 °C in cooling and heating, respectively. The studies of electric resistivity, ρ, and Seebeck coefficient, *S*, *versus* *T* for Ti_50_Ni_50−*x*_Cu*_x_* (*x* = 0~30 at.%) SMAs demonstrate that the transformation sequence exhibited in *x* = 7.5 at.% SMA is B2↔B19’ by ρ tests and is B2↔B19↔B19’ with *M*_S_’ − *M*_S_ = 15 °C by *S* tests [11]. Here, *M*_S_’and *M*_S_ are the starting transformation temperatures of B2→B19 and B19→B19’, respectively. The DSC curves for *x* = 7.5 at.% SMA also show only a B2↔B19’ peak in cooling and heating [17]. Obviously, the DMA curves for *x* = 7.5 at.% SMA, as shown in Figure 1c, cannot be used to distinguish the B19 martensite from B2↔B19’ transformation, as revealed in ρ and DSC tests.

**Figure 2:**
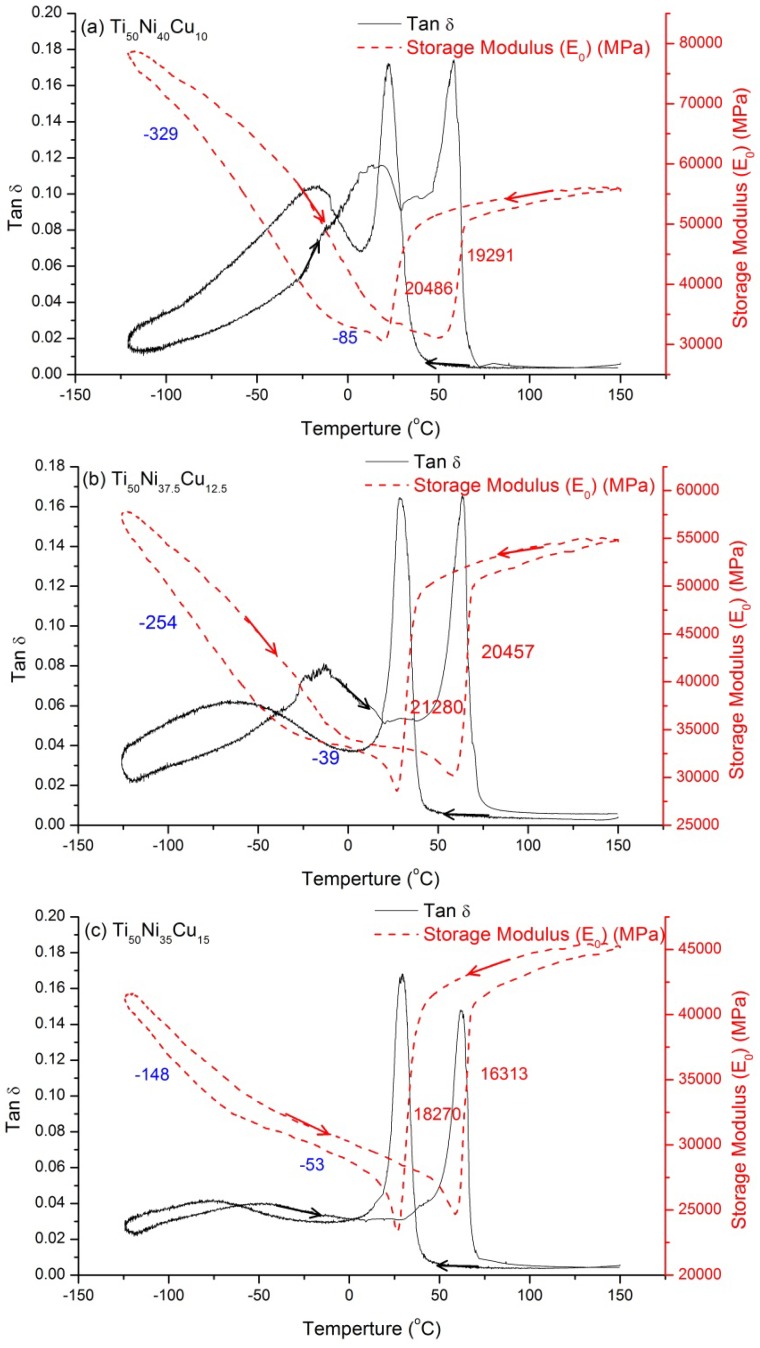
The tan δ value and storage modulus (*E*_0_) curves for Ti_50_Ni_50−*x*_Cu*_x_* SMAs. (**a**) *x* = 10; (**b**) *x* = 12.5; (**c**) *x* = 15 at.%. The blue number are the *E*_0_ slopes in B19’ and B19 martensites, and the red numbers are the magnitude of *E*_0_ softening and hardening.

**Figure 3:**
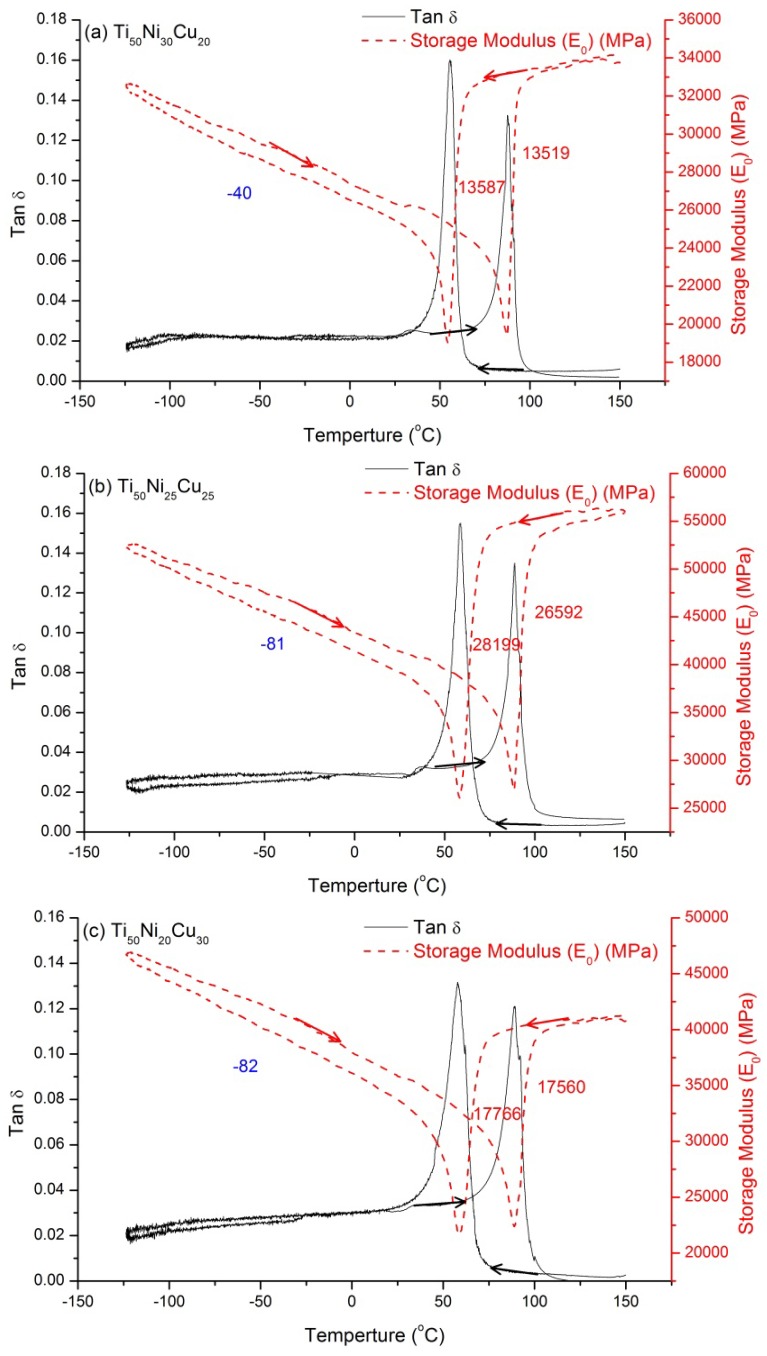
The tan δ value and storage modulus (*E*_0_) curves for Ti_50_Ni_50−*x*_Cu*_x_* SMAs. (**a**) *x* = 20; (**b**) *x* = 25; (**c**) *x* = 30 at.%. The blue number is the *E*_0_ slope in B19 martensite, and the red numbers are the magnitude of *E*_0_ softening and hardening.

Figure 2a–c and Figure 3a–c show the tan δ value *versus* *T* curves for *x* = 10, 12.5, 15 at.%, and *x* = 20, 25, 30 at.%, respectively. The former demonstrates a two-stage B2↔B19↔B19’ transformation, and the latter shows an one-stage B2↔B19 transformation, which are both consistent with those reported in previous studies [12,18,19]. Figure 2 indicates that, at the peak temperature, the tan δ value is higher but the storage modulus (*E*_0_) value is lower in B2↔B19 transformation than in B19↔B19’ transformation. At the same time, the tan δ peak is sharp for B2↔B19 transformation, but it is rather broad for B19↔B19’ transformation. This feature may have resulted from the fact that the difference of the starting and finishing transformation temperatures for B2↔B19 is much smaller than that for B19↔B19’ [11]. Figure 2 also reveals that, in cooling, B2→B19 transformation peaks have tan δ values of around 0.17, but the B2→B19’ values shown in Figure 1 are only about 0.11. Note that the transformation hysteresis of the B2↔B19’ transformation is larger than that of B2↔B19 transformation, as measured by the difference in the peaks’ temperatures shown in Figure 1 and Figure 3, respectively. In addition, no obvious relaxation peak is found in Figure 3. Therefore, from the viewpoint of the damping application at room temperature, the SMAs shown in Figure 2 are better. This is because there is a B2→B19 transformation peak appearing around the room temperature, which exhibits higher tan δ value than that for B2→B19’ transformation.

The broad peaks at around −30 °C~−70 °C for *x* = 10, 12.5, 15 SMAs shown in Figure 2 are also tested in the f range of 0.5~100Hz to identify whether they are relaxation peaks or not. Experimental results show that the *T*_p_ of these broad peaks does not shift to higher temperatures when the applied frequency is increased. Therefore, these broad peaks are not relaxation peaks but B19↔B19’ transformation peaks, for this transformation is an athermal process [20].

### 2.2. Storage Modulus Value (E_0_) versus Temperature (T)

**Figure 4:**
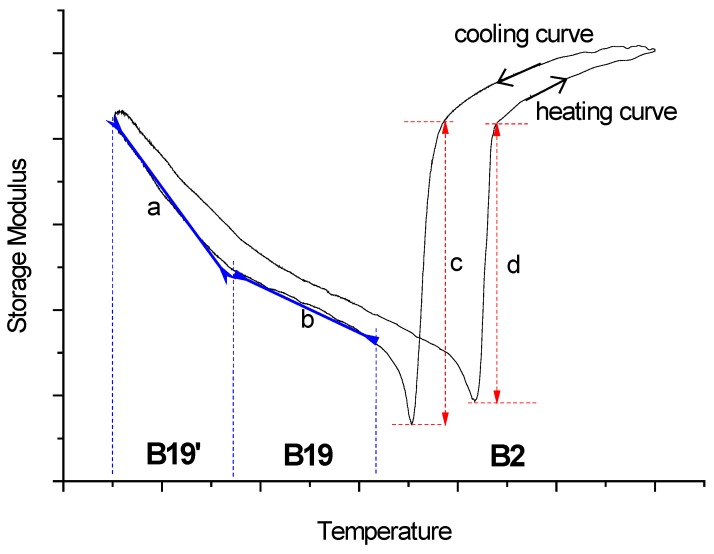
The schematic diagram for the definitions of the storage modulus (*E*_0_) softening/hardening and the slope of *E*_0_ *vs*. *T*.

It is well known that the softening of the elastic shear constant occurs in the forward martensitic transformation of SMAs [21,22]. This characteristic is also implicated in the *E*_0_ *versus* *T* curves shown in Figure 1, Figure 2 and Figure 3. From these *E*_0_ *versus* *T* curves, the values of the *E*_0_ softening/hardening and the *E*_0_ slope in B19 and B19’ martensites are measured, and they are listed in Table 1. Here, the magnitude of the *E*_0_ softening/hardening and the values of the *E*_0_ slope in B19 and B19’ martensites are defined in the schematic *E*_0_ *versus* *T* curve shown in Figure 4. Table 1 shows that the magnitude of the *E*_0_ softening in B2→B19 transformation is much larger than that in B2→B19’ transformation for *x* > 0. The same behavior also occurred in the *E*_0_ hardening associated with the reverse martensitic transformation. These features demonstrate that the *E*_0_ softening/hardening exhibited in B2↔B19 transformation is more significant than that in B2↔B19’ transformation. 

Carefully examining Figure 1, Figure 2 and Figure 3 reveals that the *E*_0_ slope in B2 phase is positive, but those in B19 and B19’ martensites are both negative. As shown in Figure 2a, for Ti_50_Ni_40_Cu_10_ SMA, the value of the *E*_0_ slope in B19 martensite is −85 MPa/°C, and that in B19’ martensite is −329 MPa/°C. This indicates that the absolute value of the *E*_0_ slope in B19’ martensite is much higher than that in B19 martensite. This characteristic may imply that the magnitude of the elastic modulus in B19 martensite is less than that in B19’ martensite. In addition, the *E*_0_ value of B19 martensite does not change much as the temperature decreases, but it changes significantly in B19’ martensite. As also can be seen from Table 1, the magnitude of *E*_0_ softening is slightly less than that of *E*_0_ hardening in B2↔B19’ transformation, but it is just the reverse in B2↔B19 transformation. This feature may be related to the insignificant *E*_0_ softening/hardening occurred in B2↔B19’ transformation.

**Table 1:** Summary of the storage modulus (*E*_0_) softening/hardening and the slope of *E*_0_ *vs*. *T* curves in B19 and B19’ martensites which are defined in the Figure 4.

Ti_50_Ni_50−*x*_Cu*_x_*, *x* (at.%)	Transformation Sequences	*E*_0_ softening (MPa)	*E*_0_ hardening (MPa)	Slope of B19 (MPa/°C)	Slope of B19’ (MPa/°C)
0	B2↔B19’	2922	1161	N/A	−166
5		3216	4700	N/A	−210
7.5		3732	4632	N/A	−211
10	B2↔B19↔B19’	20,486	19,291	−85	−329
12.5		21,280	20,457	−39	−254
15		18,270	16,316	−53	−148
20	B2↔B19	13,587	13,519	−40	N/A
25		28,199	26,592	−81	N/A
30		17,766	17,560	−82	N/A

a: the *E*_0_ slope in B19’ martensite; b: the *E*_0_ slope in B19 martensite; c: the magnitude of *E*_0_ softening; d: the magnitude of *E*_0_ hardening.

### 2.3. Strain Variation vs. Temperature (T)

**Figure 5:**
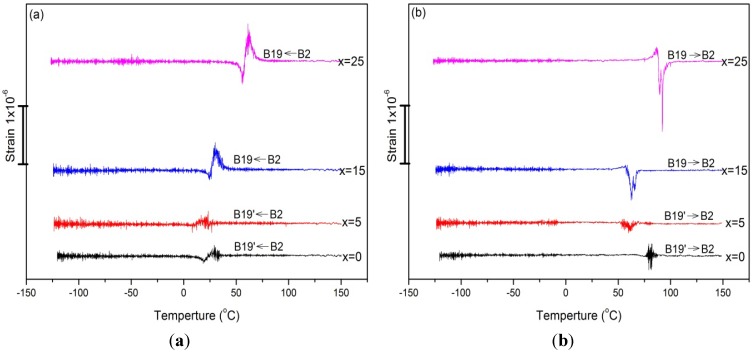
The strain variation curves for Ti_50_Ni_50−*x*_Cu*_x_* SMAs, *x* = 0, 5, 15, 25 at.% (**a**) in cooling; (**b**) in heating.

Figure 5 show the curves of strain variation *versus* temperature (*T*) for Ti_50_Ni_50−*x*_Cu*_x_* SMAs with *x* = 0, 5, 15, 25 at.% in cooling and heating, respectively. In Figure 5, the upward and downward peaks correspond to the E_0_ softening and hardening, respectively [23]. From Figure 5 and Table 1, it can be seen that the higher the E_0_ softening/hardening is, the larger the strain variation is. From Figure 5, it can be found that the magnitude of the strain variation exhibited in B2↔B19 transformation is much higher than that in B2↔B19’ transformation. This characteristic is ascribed to the fact that the former undergoes much higher *E*_0_ softening/hardening than the latter during martensitic transformation. 

### 2.4. The Damping Properties Exhibited in B2↔B19 and B2↔B19’ Transformations

It is interesting to clarify why the tan δ peak associated with B2↔B19 transformation is higher than that associated with B2↔B19’ transformation. Although the tan δ values in this study are most contributed from the transitory term (IF_Tr_), as mentioned in Section 2.1, but from the strain sweep tests [17], we found that the phase transformation term (IF_PT_) and the intrinsic term (IF_I_) associated with B2↔B19 transformation are also higher than those associated with B2↔B19’ transformation. The magnitude of the tan δ value exhibited by IF_PT_ term and that by IF_I_ term are closely related to the mobility of the phase interface between the parent phase and martensite and the twin boundary between the martensite variants. It has been reported that the twinning shear exhibited in B2→B19 transformation is smaller than that in B2→B19’ transformation [19]. This characteristic also causes the twin boundaries between B19/B19 variants to move more easily than those in between B19’/B19’ variants [19,24], and implicates that the interface between B2 and B19 phases is more mobile than that between B2 and B19’ phases. In addition, as mentioned in Section 2.3, the magnitude of the *E*_0_ softening/hardening during B2↔B19 transformation is greater than that during B2↔B19’ transformation, which can induce higher strain variation in B2↔B19 transformation than in B2↔B19’ transformation, as shown in Figure 5. It is suggested that the greater mobility of the twin boundaries (included phase interfaces) and the larger magnitude of the strain variation cause the higher tan δ value exhibited in B2↔B19 transformation than in B2↔B19’ transformation.

### 2.5. The Relaxation Peak in DMA Curves

#### 2.5.1. The Appearance of the Relaxation Peak

As mentioned in Section 2.1, the relaxation peaks are easy to obtain in Figure 1, but not in Figure 3. Ueura *et al*. [25] also found that, by DMA tests, the relaxation peak is absent in Ti_50_Ni_50−*x*_Cu*_x_* SMAs for *x* = 15, 20 and 25 at.%, but it can be seen after these SMAs were hydrogen-doped in the concentration of 0.4~12 at.% H. It has been proposed that the origin of the relaxation peak is the hydrogen atoms pinning the twinned variants during the damping test [8]. The reported studies also indicate that most of the variants in B19 martensite are not twinned [26,27,28]. Therefore, the fact that no obvious relaxation peak is observed in Figure 3 implies that the twinned variants in B19 martensite is rare. Fan *et al*. [8] reported that the relaxation peaks were observed in B19 martensite in Ti_50_Ni_34_Cu_16_ and Ti_50_Ni_30_Cu_20_ SMAs. They found that, in B19 martensite, the twins will be induced to reduce the strain energy if the specimen is slowly cooled from B2 phase. Because, in reference [8], the DMA specimen was step-cooled during the test, in which the specimen was kept isothermally for 5 min at every 5 °C, it had enough time for twins to be introduced during B2→B19 transformation, and thus the relaxation peaks were induced in B19 martensite. However, in this study, the DMA specimen was continuously cooled (3 °C/min) at a rate faster than that in Reference [8]. Therefore, it is reasonable to propose that rare twinned variants are induced in the B19 martensite, thus explaining the absence of obvious relaxation peaks in Figure 3. 

#### 2.5.2. The Activation Energy (*E*_a_) of the Relaxation Peak

**Figure 6:**
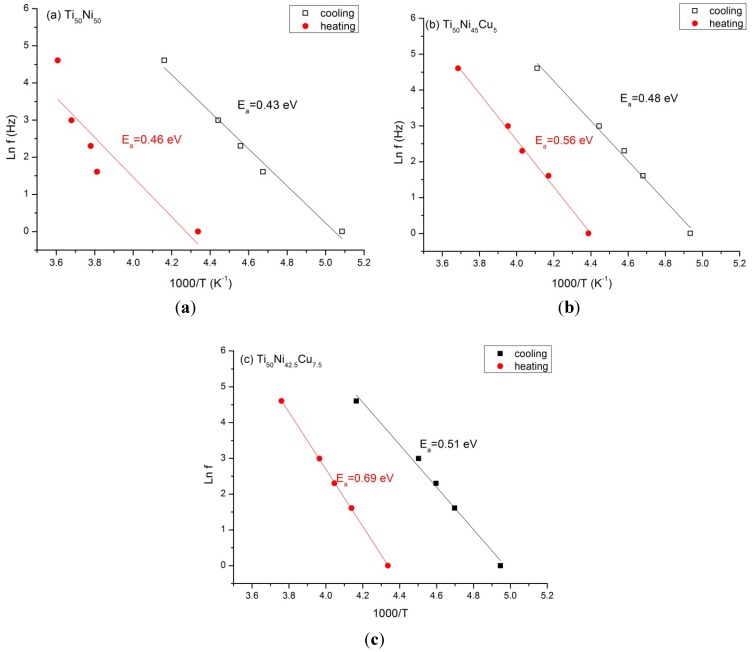
The plots of ln frequency *vs*. reciprocal relaxation peak temperature in cooling and heating processes for Ti_50_Ni_50−*x*_Cu*_x_* SMAs. (**a**) *x* = 0; (**b**) *x* = 5; (**c**) *x* = 7.5 at.%.

Figure 6a–c are the Arrhenius plots of ln *f versus* 1000/*T*_p_ for Ti_50_Ni_50−*x*_Cu*_x_*, *x* = 0, 5 and 7.5 in cooling/heating, respectively. From Figure 6, the *E*_a_ value is calculated; this value is listed in each plot and also in Table 2 for convenient comparison. Table 2 also includes the reported *E*_a_ values in Ti_50_Ni_30_Cu_15_ [25], Ti_50_Ni_34_Cu_16_ [8], Ti_50_Ni_34_Cu_20_ [8], and Ti_50_Ni_34_Cu_25_ SMAs [25]. Note that in Table 2, the relaxation peak appears in B19’ martensite for *x* ≤ 7.5, but it occurs in B19 martensite for *x* ≥ 15. From Table 2, it is clear that when *x* ≤ 7.5, the *E*_a_ value is in the range of 0.43~0.69 eV, and it increases as the Cu-content increases, whether in cooling or in heating. 

**Table 2:** The activation energy (*E*_a_) values of Ti_50_Ni_50−*x*_Cu*_x_* SMAs determined in cooling and heating processes.

Ti_50_Ni_50−*x*_Cu*_x_*, *x* (at.%)	0 ^a^	5 ^a^	7.5 ^a^	15 ^b^	16 ^c^	20 ^c^	25 ^b^
In cooling	0.43 eV	0.48 eV	0.51 eV	N/A	0.76 eV	0.67 eV	N/A
In heating	0.46 eV	0.56 eV	0.69 eV	0.68 eV	0.71 eV	0.64 eV	0.61 eV

a: Data from Figure 5 of this study; b: In Reference [25], SMAs with *x* = 15 and *x* = 25 contained 0.42 and 0.45 at.% H, respectively, after these SMAs were hydrogen-doped; c: In Reference [8], specimens were tested by dual cantilever and step cooling/heating.

However, for *x* ≥ 15, the *E*_a_ value in cooling/heating is in the range of 0.61~0.76 eV and it doesn’t change so much as the Cu-content increases. In addition, the *E*_a_ value for *x* ≥ 15 is generally larger than that for *x* ≤ 7.5. These characteristics of *E*_a_ value shown in Table 2 indicate that the effect of the hydrogen atoms pinning the twinned variants in B19’ martensite increases as the Cu-contnet increases, and this pinning effect seems to be more significant in B19 martensite than in B19’ martensite. 

## 3. Experimental Procedures

Ti_50_Ni_50−*x*_Cu*_x_* (*x* = 0, 5, 7.5, 10, 12.5, 15, 20, 25 and 30 at.%) SMAs were prepared with a vacuum arc remelter (VAR) and homogenized at 900 °C for 4 h. The titanium (purity is 99.7 wt%), nickel (purity is 99.99 wt%), and copper (purity is 99.9 wt%), totaling about 120 g, were remelted six times in an argon atmosphere, which had passed through a gas purifier to reduce its oxygen content. The weight loss during the remelting is less than 1 × 10^−4^. For Ti_50_Ni_50−*x*_Cu*_x_* SMAs with *x* ≤ 12.5 at.%, the ingots were hot rolled at 900 °C into the plates with a thickness of about 2mm by a rolling machine (DBR150 × 200 2HI-MILL, Daito Seiki Co., Hyogo, Japan), and then solution-heat-treated at 900 °C for 1 h followed by quenching in water, but those with *x* = 15~30 at.% were only solution-heat-treated at 900 °C followed by quenching in water because these SMAs are intrinsically more brittle. The surface oxide layer of the plate/ingot was removed using an etching solution of HF:HNO_3_:H_2_O =1:5:20 in volume ratio. Thereafter, the plates and the ingots were diamond-saw-cut and spark-cut, respectively, into specimens with dimensions of 40.0 × 4.8 × 1.6 mm^3^ for DMA tests. The damping properties of the specimens were measured with a TA 2980 DMA instrument equipped with a single cantilever and a liquid nitrogen cooling apparatus. The continuous cooling/heating rate was 3 °C/min, and the temperature was ranged from −130 °C to 150 °C. The applied strain and frequency were set at 7.1 × 10^−5^ and 1 Hz, respectively. From DMA tests, the curves of the tan δ, storage modulus (*E*_0_) and the strain variation values *versus* temperature (*T*) could all be determined at the same time. For calculating the activation energy (*E*_a_) of the relaxation peak, different frequencies of 0.5, 1, 5, 10, 20 and 100 Hz were employed under a constant strain of 7.1 × 10^−5^. The *E*_a_ was calculated according to Equation (1):

2π*f*·τ_0_·exp(*E*_a_/R*T*_p_) = 1 (1)

which *f* is the applied frequency, τ*_0_* is the relaxation time, R is the gas constant, and *T*_p_ is the peak temperature of the relaxation peak in absolute temperature[8].

## 4. Conclusions

DMA tests at low frequency show that Ti_50_Ni_50−*x*_Cu*_x_* SMAs exhibit B2↔B19’ transformation for *x* = 0, 5, 7.5 at.%, B2↔B19↔B19’ transformations for *x* = 10, 12.5, 15 at.%, and B2↔B19 transformation for *x* = 20, 25, 30 at.%, in which the transformation hysteresis of B2↔B19’ transformation is larger than that of B2↔B19 transformation. The tan δ, storage modulus (*E*_0_) softening/hardening, and strain variation values associated with B2↔B19 transformation are all higher than those with B2↔B19’ transformation. The larger *E*_0_ softening/hardening in B2↔B19 can induce higher strain variation in this transformation. It is suggested that the greater mobility of the twin boundaries and the larger magnitude of the strain variation cause the higher tan δ value exhibited in B2↔B19 transformation than in B2↔B19’ transformation. The *E*_0_ slope in B19’ martensite is much higher than that in B19 martensite, in which the latter is not affected so greatly by changes in temperature, but the former is significantly affected. Relaxation peaks are observed in B19’ martensite for *x* = 0, 5, 7.5 at.%, but not in B19 martensite for *x* = 20, 25, 30 at.% because only rare twinned variants are obtained in the B19 martensite. The *E*_a_ values of the relaxation peaks are calculated and compared with those reported before, and it is concluded that Ti_50_Ni_50−*x*_Cu*_x_* SMAs with higher Cu-content possess larger *E*_a_ values.
